# Adaptive Non-Stationary Fuzzy Time Series Forecasting with Bayesian Networks

**DOI:** 10.3390/s25051628

**Published:** 2025-03-06

**Authors:** Bo Wang, Xiaodong Liu

**Affiliations:** School of Control Science and Engineering, Faculty of Electronic Information and Electrical Engineering, Dalian University of Technology, Dalian 116024, China; alice_wangbo@163.com

**Keywords:** bayesian network, fuzzy time series forecasting model, adaptive learning, non-stationary time series forecasting, non-stationary fuzzy set

## Abstract

Despite its interpretability and excellence in time series forecasting, the fuzzy time series forecasting model (FTSFM) faces significant challenges when handling non-stationary time series. This paper proposes a novel hybrid non-stationary FTSFM that integrates time-variant FTSFM, Bayesian network (BN), and non-stationary fuzzy sets. We first apply first-order differencing to extract the fluctuation information of the time series while reducing non-stationarity. A novel time-variant FTSFM updating method is proposed to effectively merge historical knowledge with new observations, enhancing model stability while maintaining sensitivity to time series changes. The updating of fuzzy sets is achieved by incorporating non-stationary fuzzy sets and prediction residuals. Based on updated fuzzy sets, the system reconstructs fuzzy logical relationship groups by combining historical and new data. This approach implements dynamic quantitative modeling of fuzzy relationships between historical and predicted moments, integrating valuable historical temporal fuzzy patterns with emerging temporal fuzzy characteristics. This paper further develops an adaptive BN structure learning method with an adaptive scoring function to update temporal dependence relationships between any two moments while building upon existing dependence relationships. Experimental results indicate that the proposed model significantly outperforms benchmark algorithms.

## 1. Introduction

The advancement of modern sensor technology has facilitated the proliferation of automated data acquisition systems across diverse fields, resulting in the generation of extensive temporal observations. These data, structured as time series, document the dynamic evolution of system states, establishing a fundamental basis for predictive analysis and decision support. Numerous time series forecasting methods have been proposed [1,2]. The superior interpretability of fuzzy time series forecasting models (FTSFMs) has led to their widespread application in various fields, including financial markets [3] and wind energy [4]. FTSFMs utilize fuzzy sets to model systematic uncertainties in data, specifically imprecision and vagueness. Ref. [5] introduced the FTSFM to address time series forecasting problems. The model first fuzzifies precise original data using fuzzy sets. Next, it employs max–min composition operations on the fuzzified data to build a fuzzy relation matrix that indicates the relationships between various time points. Using historical data and the fuzzy relationship matrix, the model generates fuzzy forecast values, which are converted into precise ones. Ref. [6] improved the model of [5] by replacing the complex max–min operations with simpler arithmetic operations.

Traditional FTSFMs are optimized for stationary time series, assuming data generation from a fixed, albeit unknown, process. However, the time series generation process often changes over time in practical applications. The dynamic nature of the generation process is reflected by the data produced, which is called a non-stationary time series. The probabilistic properties of non-stationary time series change irregularly over time [7]. The development of effective fuzzy time series forecasting methodologies for such non-stationary data represents a critical research challenge.

FTSFMs struggle to keep pace with the dynamic variations of non-stationary time series, affecting forecast accuracy. To address this challenge, researchers have employed two primary approaches for reducing non-stationarity: differencing operations and decomposition methods [8,9]. Ref. [10] demonstrated that training with first-order differentiated data improves prediction accuracy compared with raw time series data. Ref. [11] proposed an intuitionistic FTSFM using the percentage of first-order differenced data between consecutive time intervals. Additionally, utilizing empirical mode decomposition methods can transform raw data into relatively more stationary multivariate time series [12,13]. Both methods decompose the original time series into multiple subsequences known as intrinsic mode functions, which possess stronger stationarity than the original non-stationary time series. However, these subsequences may incorporate future information, leading to the so-called look-ahead bias [14]. The presence of look-ahead bias can severely distort experimental results. While these stationarity transformation approaches can partially mitigate non-stationarity, they cannot completely eliminate the dynamic nature inherent in time series, necessitating fundamental improvements to FTSFMs to better accommodate temporal variations.

Several improved FTSFMs have been developed to adapt to irregular changes in non-stationary time series and reduce data non-stationarity. Ref. [5] defined the time-variant FTSFM: if the fuzzy relation matrix changes over time, it is called a time-variant FTSFM. Ref. [15] further explained the detailed implementation steps for the time-variant FTSFM. For each prediction, a fuzzy relation matrix is built using historical data from a preceding period, enabling the fuzzy relationships to change over time. The length of the historical period is referred to as the window base. Many researchers have explored time-variant FTSFMs and combined them with the aforementioned time series stabilization methods. Ref. [16] utilized differenced time series for time-variant fuzzy time series forecasting. Ref. [17] advanced the work of [16] by improving outlier handling, data fuzzification, and window base determination. Ref. [18] proposed a novel time-variant FTSFM for differenced seasonal data with a systematic search algorithm for the window base. Ref. [19] presented a time-variant FTSFM incorporating a sliding window approach [20], where the fuzzy relation matrix changes as the window slides. A propositional linear temporal logic formula is proposed to analyze the data trend in the window, thereby supporting forecasting. These time-variant FTSFMs aim to construct predictive models that can adapt to the characteristics of the latest period of data. However, neglecting the valuable information in previously trained models may result in reduced prediction quality. Since new data evolves from historical data, fully utilizing previously trained models becomes crucial for improving prediction accuracy on incoming data.

FTSFMs quantify the imprecision and vagueness within time series using fuzzy set theory. It is challenging for fixed fuzzy sets to accommodate dynamic changes in non-stationary time series. Ref. [21] proposed the definition of the non-stationary fuzzy set for dynamically adjusting fuzzy sets. A non-stationary fuzzy set is created by integrating a basic fuzzy set with a perturbation function, where the parameters of the membership function change according to the values of the perturbation function at different time points. Ref. [22] applied non-stationary fuzzy sets to FTSFMs to predict non-stationary time series with trends and scale changes, with interpolation functions serving as perturbation functions. Ref. [23] introduced a non-stationary fuzzy time series (NSFTS) forecasting model capable of handling non-stationary and heteroskedastic time series. The NSFTS model utilizes a residual-based perturbation function to adaptively adjust the membership function parameters of the basic fuzzy set, reflecting changes in the non-stationary time series. The numerical forecast is calculated by combining the midpoints of the right-hand side (RHS) of each matched rule and the membership grades of the observations. Non-stationary fuzzy sets-based forecasting models perform well in short-term non-stationary time series forecasting. However, the unchanging fuzzy relationships limit their performance in long-term non-stationary time series forecasting.

Ref. [24] proposed a time-varying FTSFM that incorporates non-stationary fuzzy sets to improve the accuracy of wind power predictions. The model handles time series variability by dividing the series into segments, each with unique membership and partition functions. Adjustments to membership function parameters employ non-stationary fuzzy set methods to suit non-stationary time series. The model retrains using the latest data window when the most recent prediction error surpasses a predefined threshold to reduce computational requirements. While [24] offers computational efficiency, its strategy of maintaining only the fuzzy relationships from the latest data window may result in the loss of valuable temporal patterns embedded in historical relationships, potentially limiting the model’s ability to capture long-term temporal relationships.

Apart from adaptively adjusting fuzzy sets in the fuzzification stage, adaptive methods have been employed to enhance other aspects of FTSFMs, thereby better accommodating the dynamic nature of non-stationary time series. Ref. [25] proposed an adaptive method that automatically modifies the order of the FTSFM based on prediction accuracy for forecasting various data. Ref. [26] applied the adaptive expectation model [27,28] to optimize the forecast outcomes of a trend-weighted FTSFM following the initial forecasts from the FTSFM. The adaptive expectation model adjusts the forecast value using the difference between it and the observation at the previous time point. Ref. [29] utilized a modified adaptive expectation model with adaptive parameters to enhance forecasting performance. Changes in the adaptive expectation model parameters indicate stock fluctuation and oscillation.

Ref. [30] introduced the Bayesian network (BN) concept. A BN represents knowledge through a probabilistic graph, with nodes denoting random variables and directed edges indicating the dependence relationships between variables. The strength of dependence between two variables is represented by their conditional probability distributions (CPDs) within a BN. The initial task when constructing a BN is to establish the BN structure that depicts the dependence relationships among variables. These relationships serve to model the causal interactions within the system. The BN structure can be manually set based on domain knowledge. In certain situations, the dependence relationships among variables are unknown and need to be inferred from data. Due to the advantages of modeling dependence relationships and handling statistical uncertainty in complex systems, several studies have applied BNs to time series forecasting. Ref. [31] initially applied BN structure learning to determine the dependence relationships in the price-earnings ratio at various time points, representing these as a BN structure. The CPDs of the time points were determined through BN parameter learning. Given historical observations at previous time points, the BN conducts probabilistic inference to generate the predicted values. Ref. [32] leveraged domain knowledge to construct the BN structure after determining the set of variables. The prediction phase initially utilized a BN to forecast the stochastic vehicular speed, followed by error compensation performed by a backpropagation neural network. Ref. [33] utilized Bayesian networks to discover direct and indirect dependence relationships across various time points in time series. Potential temporal patterns were modeled by integrating the BN structure with fuzzy logic relationships (FLRs). The study developed fuzzy empirical probability-weighted fuzzy logical relationship groups (FLRGs) to model statistical and systematic uncertainties, fully accounting for both relationships. In the above BN-based time series forecasting model, once dependence relationships are set based on all training data, they remain unchanged. This restriction reduces the model’s flexibility, which is necessary for effective time series forecasting in many situations. Ref. [34] proposed a method to construct BNs at each time point using data from a preceding period. With this approach, we can intuitively observe changes in the causal relationships within the system. Experimental results from the U.S. and Chinese stock markets indicate that the BN structure remains stable in the short term but changes over the long term. It shows that a fixed BN alone is inadequate for capturing the changing characteristics of the time series. In other words, changes in the dependencies within the BN structure can reflect the diversity of causal relationships in time series. Therefore, to enhance the BN’s ability to model complex relationships in non-stationary time series, it is necessary to develop BN structure learning methods that dynamically change based on input data.

In this study, we present a new hybrid FTSFM to enhance the accuracy of non-stationary time series forecasting. The proposed method begins by performing first-order differencing on the raw time series data. This differencing operation reduces non-stationarity while extracting information, producing a variation time series that captures fluctuations between adjacent time points. We establish the initial FTSFM using the training set of the variation time series. BN and fuzzy logical relationships (FLRs) represent the data’s temporal patterns. The BN structure visually illustrates the dependence relationships between different time points in the variation time series. At the same time, FLRs capture the fuzzy relationships between historical and forecasting moments after fuzzifying the variation time series. Uncertainty in the variation of time series is quantitatively described using FLRGs weighted by fuzzy empirical probabilities, which aggregate the membership values of corresponding FLRs within each FLRG. During the forecasting phase, we employ a sliding window approach, dividing the entire prediction dataset into multiple forecasting windows. The model remains unchanged within each window. The decision to update the existing model is based on its forecasting performance in the previous window. If no update is required, predictions are generated using the existing model; otherwise, the model is updated before predicting. When model updates are required, the proposed method employs a comprehensive updating mechanism: utilizing the training data for the existing model as old data and the actual observations from all prediction windows since the last model update as new data. The proposed method adjusts the parameters of non-stationary fuzzy sets using prediction residuals of the new data, achieving smooth transitions of fuzzy sets to respond to dynamic changes in the variation time series. The adaptive BN structure learning method employs a novel adaptive structure scoring function using old and new data, enhancing structural adaptability to new data while preserving valuable information from the dependence relationships in the existing BN. The model then reconstructs fuzzy empirical probability-weighted FLRGs using the updated BN and non-stationary fuzzy sets. After completing the model update, the framework generates predictions using the updated FLRGs and BN. The main contributions of this study are as follows:

1. We propose a novel hybrid FTSFM that integrates time-variant FTSFM, BN, and non-stationary fuzzy sets. The traditional time-variant FTSFM update strategy handles the dynamic update of fuzzy relationships. BN structure learning captures adaptive changes in temporal dependence relationships between specific time points. Non-stationary fuzzy sets address irregular changes in data imprecision. This multi-dimensional modeling strategy significantly enhances the model’s adaptability and forecasting accuracy for non-stationary time series.
2. We develop an adaptive BN structure updating method with a novel dynamic scoring mechanism. The proposed method enables continuous refinement of temporal dependence relationships while preserving crucial historical patterns, thereby achieving an optimal balance between stability and adaptability in temporal relationship modeling.
3. We introduce a novel non-stationary fuzzy set approach that enhances existing methods through an innovative residual-based perturbation mechanism. This perturbation function enables each fuzzy set to share the impact of prediction residuals through distinct displacement degrees, facilitating smooth transitions of fuzzy sets. It ensures the model’s sensitivity to changes in the vagueness of non-stationary time series while enhancing its stability.

The remaining sections of this paper are structured as follows. In Section 2, we provide a detailed explanation of FTSFMs and BNs serving as the basis for the proposed algorithm. Section 3 provides an in-depth description of the proposed FTSFMs with BNs in non-stationary environments. Experimental result analyses are presented in Section 4. Section 5 concludes the paper.

## 2. Preliminaries

In this section, basic definitions of FTSFMs, non-stationary fuzzy sets, and BNs are briefly presented.

### 2.1. Basic Concepts of Fuzzy Time Series Model

Let *U* be the universe of discourse. A fuzzy set *A* on *U* is expressed as

(1) $$A=\int_{u\in U}\mu_{A}\left(u\right)/u,$$

where $\mu_{A}$ denotes the membership function of *A*, $\mu_{A}:U\mapsto \left[0,1\right]$. $\mu_{A}\left(u\right)$ is the membership grade of u∈U, and $\mu_{A}\left(u\right)\in \left[0,1\right]$. Let the parameters of $\mu_{A}$ be $p_{1},\ldots ,p_{m}$. $\mu_{A}\left(u\right)$ can be expressed as $\mu_{A}\left(u,p_{1},\ldots ,p_{m}\right)$.

A triangular fuzzy set *A* takes the triangular function as the underlying membership function. Denote the lower, midpoint, and upper values of the triangle as a,b, and *c*, respectively. The membership function is defined as

(2) $$\mu_{A}\left(u,a,b,c\right)=\left\{\begin{array}{cc} (u-a)/(b-a),\; & a\leq u\leq b\\ (c-u)/(c-b),\; & b\leq u\leq c\\ 0,\; & \mathrm{else}.\; \end{array}\right.$$

Suppose a time series $Y=\{y_{t}|t\in T\}$ is given with $y_{t}\in R$. Fuzzy sets $A_{1},...,A_{I}$ are defined on the universe of discourse *U*. Membership grades of $y_{t}$ belong to the fuzzy sets formed from the fuzzified data $f_{t}=\left[\mu_{A_{1}}\left(y_{t}\right),\ldots ,\mu_{A_{I}}\left(y_{t}\right)\right]$ at the moment *t*. *F* is the fuzzy time series defined on *Y* with the collection of $f_{t}$.

When $f_{t}$ results from $f_{t-h},...,f_{t-1}$, FLRs represent the fuzzy relationship between the antecedent moments t−h,...,t−1 and the consequent moment *t*. An FLR for $f_{t-h},...,f_{t}$ has the format $A_{i}^{t-h},...,A_{i}^{t-1}\to A_{i}^{t}$, where the membership grade of $y_{t-k}$ on the fuzzy set $A_{i}^{t-k}$ (k=0,...,h) is greater than zero. Each combination of ${\left\{f_{t-k}\right\}}_{k=h}^{0}$ can yield multiple FLRs, given that each $f_{t-k}$ may comprise multiple elements with non-zero membership grades. FLRs that have the same left-hand side constitute an FLRG denoted as $A_{i}^{t-h},...,A_{i}^{t-1}\to A_{k_{1}},A_{k_{2}},\ldots$.

### 2.2. Non-Stationary Fuzzy Set

Ref. [21] introduced non-stationary fuzzy sets with the help of the non-stationary membership function and the perturbation function. The non-stationary membership function reflects the temporal variability present in membership functions. The perturbation function calculates the dynamic component of function parameters when the membership function changes. Non-stationary fuzzy sets reflect data change through positional shifts, changes in width, and noise-induced variations in the membership grade.

A non-stationary fuzzy set $\dot{A}$ is denoted as follows:

(3) $$\dot{A}=\int_{t\in T}\int_{u\in U}\mu_{\dot{A}}\left(t,u\right)/u/t,$$

where *T* contains a series of time points and $\mu_{\dot{A}}\left(t,u\right):T\times U\mapsto \left[0,1\right]$ is the non-stationary membership function. $\mu_{\dot{A}}\left(t,u\right)$ changes over time in the time interval *T*, which can be expressed as

(4) $$\mu_{\dot{A}}\left(t,u\right)=\mu_{A}\left(u,p_{1}\left(t\right),\ldots ,p_{m}\left(t\right)\right).$$

$p_{i}\left(t\right)=p_{i}+c_{i}b_{i}\left(t\right)$ with a time-variant perturbation function $b_{i}\left(t\right)$ and a constant $c_{i}$ for i=1,…,m.

### 2.3. Bayesian Network

Bayesian networks have demonstrated their remarkable effectiveness for complex data-analysis problems [35]. The BN is a member of probabilistic graphical models. BNs can represent the latent patterns within data by incorporating a set of variables and their dependence in the form of directed acyclic graphs. A BN includes $N_{v}$ nodes representing random variables. Values of these nodes are possible observations of the variables. BNs visually represent conditional independence through directed acyclic graphs. A BN also utilizes an adjacency matrix *G* representing the edges between variables, with $G_{ij}=1$ indicating the directed dependence relationship from the *i*-th node to the *j*-th node. The CPD $P(X_{i}|Pa_{i})$ for the *i*-th node represents the strength of the dependence between the *i*-th node and its parent nodes $Pa_{i}$. $P(X_{i}=x_{i}|Pa_{i}=pa_{j})$ denotes the probability of the value $x_{i}$ of the *i*-th node given the *j*-th set of observations $pa_{j}$ of parent nodes $Pa_{i}$. The directed acyclic graph of a BN factorizes the joint probability distribution over variables $X=\{X_{1},\ldots ,X_{N_{v}}\}$:

(5) $$P\left(X_{1},\ldots ,X_{N_{v}}\right)=\prod_{i=1}^{N_{v}}P\left(X_{i}|Pa_{i}\right)$$

Representing dependence relationships between variables in a BN requires acquiring the directed acyclic graph structure through learning methods, typically achieved using BN structure learning techniques. Apart from structure learning, BN learning also includes parameter learning. Parameter learning involves the determination of CPD parameters, while structure learning aims at generating the adjacency matrix to discover dependence relationships between variables. Structure and parameter learning are interdependent, as parameter learning needs the BN structure to identify the parents of each node before computing CPDs. Moreover, parameter learning is essential for evaluating the matching degree of a candidate network structure and the data.

Data-driven BN structure learning methods principally fall into two categories: constraint-based algorithms and score-based algorithms. The core idea of the latter is to explore the space of all potential directed acyclic graphs using a search strategy to select the optimal graph based on the values yielded from a scoring function on the gathered data. The present research employs a score-based structural learning method using the hill-climbing search method and Bayesian information criterion (BIC) as the score function [36]. The BIC scoring function offers the benefit of decomposability and clear intuitiveness. The BIC score approximates the marginal likelihood function as follows:

(6) BIC(G|D)=logP(G|D)−f(G|D),

where logP(G|D) is the logarithm likelihood of the graph structure *G* given the dataset *D*. The CPD parameters are determined by the maximum likelihood estimation algorithm. The penalty term $f\left(G|D\right)=N_{params}/2\cdot \log N_{D}$ helps prevent overfitting. $N_{params}$ represents the count of parameters in all CPDs within the BN. The dataset *D* contains $N_{D}$ data instances. The BIC function for a BN can be factorized as

$$BIC\left(G|D\right)=\sum_{i}BIC\left(X_{i}|Pa_{i},D\right)=\sum_{i}\log P\left(X_{i}|Pa_{i},D\right)-f\left(X_{i}|D\right),$$

where $BIC(X_{i}|Pa_{i},D)$ is the BIC score of the variable $X_{i}$ and $f\left(X_{i}|D\right)=N_{params_{i}}/2\cdot \log N_{D}$.

The hill-climbing method is a widely used search algorithm. The search starts with an initial model, which can either be an empty graph or a specific graph. Each search iteration produces candidate models derived from a single modification of the current model. When applying the hill-climbing algorithm to BN structure learning, the model with superior performance is preserved by comparing the scores of each candidate model and the current model via the scoring function. Operations such as adding, deleting, or reversing an edge generate candidate models during each iteration. As the BIC score function is decomposable, the comparison between candidate models and the current model can focus solely on the score of their dissimilar segments. Combining domain knowledge with a data-driven learning method is a common practice in BN learning. This paper models the BN structure by blending temporal adjacency relationships as domain knowledge with raw time-series data. A detailed description of the hill climbing and BIC-based BN structure learning method is introduced in [33].

## 3. Proposed Method

This section introduces a novel FTSFM, abbreviated as TV-NS-BN-PWFTS (Time-Variant Non-Stationary Bayesian Network-based Probabilistic Weighted Fuzzy Time Series), which incorporates adaptive structure learning of BN and non-stationary fuzzy set into the time-variant FTSFM based on the fundamental concepts mentioned earlier.

**Figure 1:**
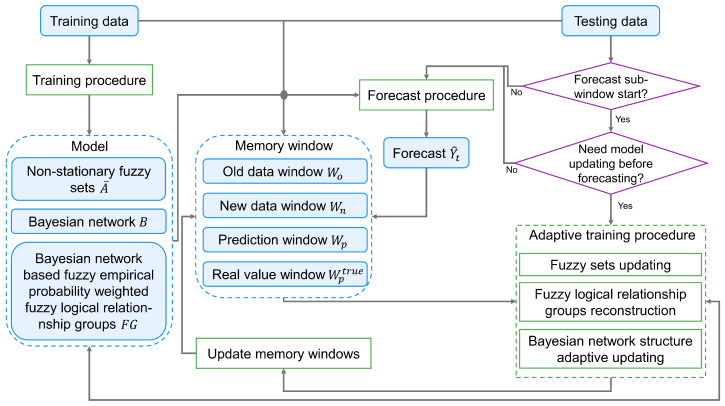
The flow chart of the proposed model.

The proposed methodology consists of two primary phases: initial model construction during training (Section 3.1), followed by a dynamic forecasting process utilizing a sliding window approach (Section 3.2). During the forecasting phase, the model continuously monitors prediction residuals from the most recent window. When these residuals exceed a preset threshold value, the model updates using data from existing available prediction windows before generating predictions for the current window. Figure 1 depicts the workflow of the proposed model. The following are descriptions of the various stages of the proposed method.

### 3.1. Training Procedure

Traditional FTSFMs focus on capturing the intrinsic patterns in time series by establishing fuzzy relationships and using fuzzy sets and membership degrees to qualitatively and quantitatively describe systematic uncertainty. The Bayesian network-based probabilistic weighted fuzzy time series (BN-PWFTS) model proposed by [33] models temporal patterns in time series by combining BN and FLRs. BN fully considers direct and indirect dependence relationships between different time points, thus incorporating additional information about potential patterns beyond fuzzy relationships, providing more information for modeling the uncertainty of future time series values. To model systematic and statistical uncertainties, BN-PWFTS defines BN-based probabilistic weighted FLRGs. The probabilistic weights of elements in an FLRG are calculated as fuzzy empirical conditional probabilities using dependence relationships and membership degrees. The weights assigned to the antecedent and consequent components of FLRGs are determined by systematically incorporating the fuzzy empirical conditional probabilities of their constituent elements, leveraging the established dependency relationships. Although BN-PWFTS makes predictions by modeling interrelationships and uncertainties in time series, it lacks the ability to adapt to non-stationary changes in the time series. To address this limitation, we propose an enhanced model, TV-NS-BN-PWFTS, which not only preserves the advantages of BN-PWFTS but also effectively handles non-stationarity in time series data. In order to develop an effective initial model that can adequately capture both the intricate patterns and underlying uncertainties within time series data, we have adopted the training methodology from the fuzzy time series forecasting model proposed by [33]. In the training procedure, first-order differencing is conducted to decrease the non-stationarity of the time series, producing a differenced time series that captures variations between consecutive observations. Subsequently, the BN-PWFTS training process is employed to capture the intricate temporal relationships and uncertainties in the differenced time series. Algorithm 1 contains a detailed description of the training procedure.

**Algorithm 1** Training procedure
**Input:** $Y_{t}^{tr}$—the original value at the time point *t* in the training dataset, ω—the order of FTSFM.**Output:** FG—BN-based probabilistic weighted fuzzy logical relationship groups (BN-PWFLRGs), *B*—the trained BN, $\bar{A}$—fuzzy sets, *Z*—partition functions.
*// Non-stationary time series stabilization*
  1:Compute the variation in time series between two adjacent time points YD as $YD_{t}=Y_{t}^{tr}-Y_{t-1}^{tr}$.
*// Fuzzy set construction*
  2:Define *U* as the universe of discourse of YD, and split *U* into *I* equal-length intervals $\left\{U_{i}\right\}$ with midpoints $\left\{m_{i}\right\}$. The fuzzy sets $\bar{A}=\left\{A_{i}\right\}$ are built on *U* with membership functions $\mu_{A_{i}}\left(\right)$, where 1≤i≤I.
*// Time series fuzzification*
  3:Generate the fuzzified time series $F=\left\{f_{t}\right\}=\left\{\left[\mu_{A_{1}}\left(YD_{t}\right),\ldots ,\mu_{A_{I}}\left(YD_{t}\right)\right]\right\}$. $0\leq \mu_{A_{i}}\left(YD_{t}\right)\leq 1$.
*// Fuzzy relationship modeling*
  4:Generate FLRs from the fuzzified data with the format $A_{i}^{t-\omega },...,A_{i}^{t-1}\to A_{i}^{t}$. Multiple FLRs may be generated for some given set of time points.  5:Generate FLRGs $FG=\left\{FG_{A_{i_{lhs}}}\right\}=\left\{A_{i}^{t-\omega },...,A_{i}^{t-1}\to A_{k_{1}},A_{k_{2}},\ldots \right\}$ by gathering all FLRs with the same left-hand side (LHS) $A_{i_{lhs}}=A_{i}^{t-\omega },...,A_{i}^{t-1}$.
*// BN structure learning for dependence relationship modelling*
  6:YD is transformed into a ω+1-variate dataset $C=\{c_{1},c_{2},...,c_{T_{1}-\omega }\}$. $c_{t}=\left[YD_{t-\omega },...,YD_{t}\right]$ represents a series of observations of ω historical time points and a prediction moment *t*.  7:Taking observations of the ω+1 moments as variable values, construct dependence relationships between these moments as a BN *B* using a hill-climbing algorithm and BIC (Equation (6)) based BN structure learning method.
*// BN-based fuzzy empirical probability calculation*
  8:**for** 0≤k≤ω **do**  9:   Compute the fuzzy empirical conditional probability $P(A_{i}^{t-k}|A_{j}^{Pa_{t-k}})$ of the moment t−k in an FLRG. $P(A_{i}^{t-k}|A_{j}^{Pa_{t-k}})$ is determined below:(7)$$P\left(A_{i}^{t-k}|A_{j}^{Pa_{t-k}}\right)=\frac{\sum_{YD_{t-k},pa_{t-k}}\mu_{A_{i}}\left(YD_{t-k}\right)\mu_{A_{j}^{Pa_{t-k}}}\left(pa_{t-k}\right)}{\sum_{i}\sum_{YD_{t-k},pa_{t-k}}\mu_{A_{i}}\left(YD_{t-k}\right)\mu_{A_{j}^{Pa_{t-k}}}\left(pa_{t-k}\right)},$$ where $A_{j}^{Pa_{t-k}}$ is the *j*-th fuzzy set group for the parent moments of t−k identified by the learned *B*. $pa_{t-k}$ is the set of observations in YD for the parent moments. $\mu_{A_{j}^{Pa_{t-k}}}\left(pa_{t-k}\right)$ denotes the product of the membership degrees of the parent moments $\prod_{s\in Pa_{t-k}}\mu_{A_{i_{s}}}\left(pa_{s}\right)$.10:
**end for**
11:Compute the fuzzy empirical probability of the LHS of an FLRG with $A_{i_{lhs}}$ based on the fuzzy empirical conditional probabilities of historical time points according to Equations (5) and (7).(8)$$P\left(A_{i_{lhs}}\right)=\prod_{k=\omega }^{1}P\left(A_{i}^{t-k}|A_{j}^{Pa_{t-k}}\right).$$12:Compute the fuzzy empirical probabilities of the RHS of an FLRG with $A_{i_{lhs}}$ according to Bayes rule(9)$$P\left(A_{i}^{t}|A_{i_{lhs}}\right)=\frac{P(A_{i}^{t},A_{i_{lhs}})}{P(A_{i_{lhs}})}.$$13:Calculate the partition functions $Z=\{Z_{A_{i_{lhs}}}\}$(10)$$Z_{A_{i_{lhs}}}=\sum_{YD_{t-\omega },...,YD_{t-1}\in U}\prod_{k=\omega }^{1}\mu_{A_{i}^{t-k}}\left(YD_{t-k}\right).$$14:Assign weights of the LHS and RHS to construct the BN-PWFLRG $FG_{A_{i_{lhs}}}$ with the format as $P\left(A_{i_{lhs}}\right)\cdot A_{i_{lhs}}\to P\left(A_{1}^{t}|A_{i_{lhs}}\right)\cdot A_{1}^{t},\ldots ,P\left(A_{I}^{t}|A_{i_{lhs}}\right)\cdot A_{I}^{t}$.15:**return** the set of BN-PWFLRGs $FG=\{FG_{A_{i_{lhs}}}\}$, the learned BN *B*, the fuzzy sets $\bar{A}$, and the partition functions $Z=\{Z_{A_{i_{lhs}}}\}$.


### 3.2. Forecasting Procedure

In many non-stationary time series forecasting scenarios, newly arrived data often deviates from previously observed patterns in historical data, presenting significant challenges to traditional FTSFM. To address this limitation, we propose a novel forecasting procedure that incorporates a dynamic updating mechanism. This approach allows the model to continuously adapt to the the uncertainty and temporal patterns within new data, ensuring accurate predictions even when the underlying characteristics change over time. The following section first introduces the dynamic updating mechanism for non-stationary fuzzy sets and BN structure, followed by a detailed discussion of the complete forecasting procedure.

#### 3.2.1. Non-Stationary Fuzzy Set Updating with New Perturbation Function

In non-stationary time series forecasting, traditional fuzzy sets struggle to adapt to dynamic changes in data distribution. Ideally, once the perfect model captures all information in the data, its prediction residuals should exhibit a standard normal distribution. Ref. [23] therefore designed perturbation functions based on the mean and variance of residuals to adjust non-stationary fuzzy set parameters, driving prediction residuals toward a standard normal distribution. However, the uniform adjustment with residual means ignores the different contributions of individual fuzzy sets to the residuals. We propose an improved residual-based non-stationary fuzzy set perturbation function. The proposed non-stationary fuzzy sets are built on the first-order differencing time series instead of the original time series to capture additional non-stationary features. Unlike [23], our method updates the fuzzy sets only when the model’s prediction performance for the current time period falls below a threshold. The proposed perturbation function employs a uniform distribution strategy to assign the residual mean to each fuzzy set, enabling gradual adjustments from the original position towards the new arrival data. This strategy maintains the valuable historical information within the existing fuzzy sets while promptly capturing the time series’ evolving characteristics.

This paper designs a new non-stationary fuzzy set parameter adjustment method based on triangular membership function Equation (2). For any non-stationary fuzzy set $A_{i}$ built on the differenced time series, its perturbed membership function is expressed as $\mu_{A_{i}}\left(YD_{t},p\left(a_{i},b_{i},c_{i},d_{i},s_{i}\right)\right)$, where the perturbation function $p(a_{i},b_{i},c_{i},d_{i},s_{i})$ is defined as:

(11) $$p\left(a_{i},b_{i},c_{i},d_{i},s_{i}\right)=\left\{a_{i}+d_{i}-s_{i}/2,b_{i}+d_{i},c_{i}+d_{i}+s_{i}/2\right\},$$

where $d_{i}$ and $s_{i}$ denote the displacement and scaling factors of fuzzy set $A_{i}$, respectively, both computed based on prediction residuals. Let the residual mean be $\bar{E}$ and the variance be $\sigma_{E}$. The displacement parameter $d_{i}$ for fuzzy set migration based on residual statistics is calculated as follows:

(12) $$d_{i}=\left\{\begin{array}{cc} \frac{i\cdot \bar{E}}{I}+i\frac{2\sigma_{E}}{I-1}-\sigma_{E}, & \mathrm{if}\;\bar{E}\geq 0\\ \frac{\left(I-i\right)\cdot \bar{E}}{I}+i\frac{2\sigma_{E}}{I-1}-\sigma_{E}, & \mathrm{if}\;\bar{E}<0. \end{array}\right.$$

This design enables fuzzy sets to progressively approach the zero-mean residual direction through $\frac{i\cdot \bar{E}}{I}$ as *i* increases while incorporating boundary scaling information via $i\cdot 2\sigma_{E}/\left(I-1\right)-\sigma_{E}$. The coverage range of fuzzy sets is modulated by the scaling parameter

(13) $$s_{i}=\left|d_{i-1}-d_{i+1}\right|,$$

which ensures smooth transitions between adjacent fuzzy sets. The proposed membership function parameter adjustment mechanism enables fuzzy sets to dynamically adapt to the non-stationary characteristics of the time series.

#### 3.2.2. BN Structure Adaptive Updating

Existing FTSFMs exhibit significant limitations when handling non-stationary time series: they either employ fixed FLRs [23] or exclusively utilize the latest data for constructing FLRs. Both strategies fail to achieve effective long-term prediction due to their inability to fully leverage the crucial temporal pattern information embedded in historical data. To overcome this limitation, we propose a novel time-variant FTSFM updating strategy. Once the initial FTSFM is established during training, the model undergoes dynamic updates at irregular intervals during the prediction phase. We assume that all data used to train the current model are old data $D_{old}$, and the actual values of the periods predicted by the current model are new data $D_{new}$. The model update regarding temporal patterns includes two parts: (1) After updating non-stationary fuzzy sets, FLRs and FLRGs are reconstructed using both $D_{old}$ and $D_{new}$, retaining historical temporal patterns to some extent while capturing pattern changes in new data. (2) The BN structure, containing dependence relationships for each time point, is updated through a hill-climbing-based structure learning method with an adaptive BIC. This method adaptively adjusts the learning process to balance the influence of new and old data. Additionally, updating based on the existing BN structure ensures the preservation of valuable historical dependence information. The BN updating process is summarized in Algorithm 2.

**Algorithm 2** BN structure adaptive updating
**Input:** $D_{old}$—the data used to train the current FTSFM, $D_{new}$—real observations of the forecasting sub-windows predicted by the current FTSFM, *B*—the current BN, η—weighting parameter in the adaptive BIC, ω—the order of FTSFM.**Output:** Updated BN $B^{*}$.  1:Initialize variables $X=\{X_{1},...,X_{\omega +1}\}$ representing the time points in FLRG  2:

$B^{*}\leftarrow B$

  3:Compute the score $S_{old}$ of the initial BN structure *B* with the adaptive BIC score function $BIC_{a}\left(\right)$(14)$$\begin{array}{cc} S_{old}=BIC_{a}\left(B|D_{old},D_{new},\eta \right)= & \eta \left[\log P\left(B|D_{old}\right)-\frac{N_{params}}{2}\log\left|D_{old}\right|\right]\;\\ & +\left(1-\eta \right)\left[\log P\left(B|D_{new}\right)-\frac{N_{params}}{2}\log\left|D_{new}\right|\right] \end{array}$$  4:**while** not converged **do**  5: **for** each possible edge operation op∈{add,delete,reverse} **do**  6:  **for** each possible edge $e=X_{i}\to X_{j}$ for operation op **do**  7:   $B^{\prime }\leftarrow$ Apply op with edge *e* in *B*  8:   **if** $B^{\prime }$ is acyclic **then**  9:    $\Delta_{op}^{e}\leftarrow BIC_{a}\left(B^{\prime }|D_{old},D_{new},\eta \right)-BIC_{a}\left(B|D_{old},D_{new},\eta \right)$,10:    **if** $\Delta_{op}^{e}>0$ **then**11:     $S_{best}\leftarrow BIC_{a}\left(B^{\prime }|D_{old},D_{new},\eta \right)$12:     $B_{best}\leftarrow B^{\prime }$13:    **end if**14:   **end if**15:  **end for**16: **end for**17: **if** $S_{best}>S_{old}$ **then**18:    $B^{*}\leftarrow B_{best}$19:    $S_{old}\leftarrow S_{best}$20: **else**21:  **return** $B^{*}$22: **end if**23:
**end while**



#### 3.2.3. Integrated Forecasting Framework

The forecasting process employs a dynamic prediction framework that adaptively updates the model based on prediction residuals and historical data. The model’s performance in the previous sub-window is evaluated using the mean absolute scaled error (MASE) before each new forecasting sub-window. The MASE metric is defined as follows:

(15) $$\mathrm{MASE}=\frac{\frac{1}{l_{r}}\sum_{t=1}^{l_{r}}\left|Y_{t}-{\hat{Y}}_{t}\right|}{\frac{1}{l_{r}-1}\sum_{t=2}^{l_{r}}\left|Y_{t}-Y_{t-1}\right|},$$

where $Y_{t}$ is the actual value, ${\hat{Y}}_{t}$ is the predicted value at time point *t*, and $l_{r}$ is the length of the prediction sub-window. MASE offers a scale-independent measure of prediction accuracy. MASE<1 indicates better performance compared with the naive approach of using the previous observation as the prediction. A lower MASE value indicates better predictive performance of the model. The model is updated when the MASE surpasses a predefined threshold θ, which signifies that the current model’s prediction accuracy on the latest prediction window is unsatisfactory. The forecasting procedure is detailed in Algorithm 3, including the numerical prediction generation process in Algorithm 4.

**Algorithm 3** Forecasting procedure
**Input:** $Y_{t}$—the original value at the time point *t* in the testing dataset, *B*—the initial trained BN, FG—BN-based probabilistic weighted fuzzy logical relationship groups (BN-PWFLRGs), ω—the order of FTSFM, θ—the threshold for model update, $l_{o}$—the length of old data memory window, $l_{n}$—the length of new data memory window, $l_{p}$—the length of prediction window, $\bar{A}$—fuzzy sets, *Z*—partition functions, $Y^{tr}$—the training dataset.**Output:** $\hat{Y}$—all predicted values.  1:Initialize the old data memory window $W_{o}$ by the last $l_{o}$ samples of $Y^{tr}$  2:Initialize the new data memory window $W_{n}$, the prediction result memory window $W_{p}$, and the true value memory window $W_{p}^{true}$ as ∅  3:**for** t=1 to $N_{te}$ **do**
*// Check if need update model before forecasting the initial point in each forecasting sub-window*
  4: **if** t=1 **then**  5:  Generate the prediction ${\hat{Y}}_{t}$ with $Y_{t-\omega },...,Y_{t-1}$, *Z*, $\bar{A}$ and FG (Algorithm 4)  6:  $W_{p}\leftarrow W_{p}\cup \left\{{\hat{Y}}_{t}\right\}$  7: **end if**  8: **if** $t\%l_{r}==1$ and t>1 **then**  9:   $W_{p}^{true}\leftarrow W_{p}^{true}\cup \left\{Y_{t-1}\right\}$, $W_{n}\leftarrow W_{n}\cup \left\{Y_{t-1}\right\}$10:   **if** $\mathrm{MASE}(W_{p}^{true},W_{p})\geq \theta$ **then**11:   $D_{old}\leftarrow W_{o}$, $D_{new}\leftarrow W_{n}$12:   Adaptively update the BN *B* with $D_{old}$, $D_{new}$, ω, and η to obtain the updated BN $B^{*}$ (Algorithm 2) and $B\leftarrow B^{*}$13:   Update the non-stationary fuzzy sets $\bar{A}$ with the perturbation function Equations (11)–(13)14:   Reconstruct BN-PWFLRGs $FG=\{FG_{A_{i_{lhs}}}\}$ with the format as $P\left(A_{i_{lhs}}\right)\cdot A_{i_{lhs}}\to P\left(A_{1}^{t}|A_{i_{lhs}}\right)\cdot A_{1}^{t},\ldots ,P\left(A_{I}^{t}|A_{i_{lhs}}\right)\cdot A_{I}^{t}$ by $D_{old}$, $D_{new}$, the updated $\bar{A}$ and *B* (Equations (7)–(9)).15:   Recalculate the partition functions *Z* based on $D_{old}$, $D_{new}$, the updated $\bar{A}$ and *B* (Equation (10)).16:   Generate the prediction ${\hat{Y}}_{t}$ with $Y_{t-\omega },...,Y_{t-1}$, *Z*, $\bar{A}$ and FG (Algorithm 4)17:   $W_{o}\leftarrow$ the last $l_{o}$ samples of $W_{o}\cup W_{n}$, $W_{n}\leftarrow \emptyset$, $W_{p}^{true}\leftarrow \emptyset$, $W_{p}\leftarrow \left\{{\hat{Y}}_{t}\right\}$18:  **else**19:   Generate the prediction ${\hat{Y}}_{t}$ with $Y_{t-\omega },...,Y_{t-1}$, *Z*, $\bar{A}$ and FG (Algorithm 4)20:   $W_{p}\leftarrow W_{p}\cup \left\{{\hat{Y}}_{t}\right\}$21:  **end if**22: **else**23:  Generate the prediction ${\hat{Y}}_{t}$ with $Y_{t-\omega },...,Y_{t-1}$, *Z*, $\bar{A}$ and FG (Algorithm 4)24:  $W_{n}\leftarrow W_{n}\cup \left\{Y_{t-1}\right\}$, $W_{p}\leftarrow W_{p}\cup \left\{{\hat{Y}}_{t}\right\}$, $W_{p}^{true}\leftarrow W_{p}^{true}\cup \left\{Y_{t-1}\right\}$25: **end if**26:
**end for**
27:**return** $\hat{Y}=\left\{{\hat{Y}}_{1},\ldots ,{\hat{Y}}_{N_{te}}\right\}$.


**Algorithm 4** Generate prediction for time point *t*
**Input:** $Y_{t-\omega },...,Y_{t-1}$—historical data, $\bar{A}$—fuzzy sets, FG—BN-based probabilistic weighted fuzzy logical relationship groups (BN-PWFLRGs), *Z*—partition functions.**Output:** ${\hat{Y}}_{t}$—predicted value.1:Generate $YD_{t-l}=Y_{t-l}-Y_{t-l-1}$ (l=1,...,w) by first-order differencing.2:Fuzzify $YD_{t-l}$ into $F_{t-l}=\left\{A_{i}^{t-l}|\mu_{A_{i}}\left(YD_{t-l}\right)>0\right\}$ based on $\bar{A}$, l=1,...,w.3:Construct each possible pair of $F_{t-\omega },...,F_{t-1}$ denoted by $A_{i_{lhs}}=\left\{A_{i}^{t-\omega },...,A_{i}^{t-1}\right\}$ as the LHS of an FLRG4:Locate the BN-PWFLRG $FG_{A_{i_{lhs}}}\in FG$ that has the same LHS $A_{i_{lhs}}$ as the active FLRG for each $A_{i_{lhs}}$.5:Calculate the expectation $E(mp_{A_{i_{lhs}}})$ of midpoints of fuzzy sets on the RHS of $FG_{A_{i_{lhs}}}$ according to Equations (8) and (9):(16)$$E\left(mp_{A_{i_{lhs}}}\right)=\sum_{j\in I}P\left(A_{j}|A_{i_{lhs}}\right)\cdot mp_{j}.$$6:Calculate the prediction based on all active FLRGs:(17)$${\hat{YD}}_{t}=\sum_{i_{lhs}}\frac{P(YD_{t-\omega },...,YD_{t-1}|A_{i_{lhs}})}{\sum_{i_{lhs}}P\left(YD_{t-\omega },...,YD_{t-1}|A_{i_{lhs}}\right)}E\left(mp_{A_{i_{lhs}}}\right),$$
where $P\left(YD_{t-\omega },...,YD_{t-1}|A_{i_{lhs}}\right)=P\left(A_{i_{lhs}}\right)\mu_{A_{i_{lhs}}}\left(YD_{t-\omega },...,YD_{t-1}\right)/Z_{A_{i_{lhs}}}$ according to Equation (10). $\mu_{A_{i_{lhs}}}\left(YD_{t-\omega },...,YD_{t-1}\right)$ is the product of membership degrees of $YD_{t-\omega },...,YD_{t-1}$ on fuzzy sets in $A_{i_{lhs}}$.7:Inverse differencing to obtain the final numerical prediction ${\hat{Y}}_{t}\leftarrow Y_{t-1}+{\hat{YD}}_{t}$.8:**return** ${\hat{Y}}_{t}$.


## 4. Experiments

### 4.1. Experimental Design

This section verifies the superiority of the proposed model in forecasting non-stationary time series. First, an overview of datasets and evaluation metrics is provided [23,37,38,39]. Next, we benchmark the proposed model against various non-stationary FTSFMs and state-of-the-art forecasting models in batch mode. All software is executed on a Windows 11 desktop machine of intel core i5-13400F with 16 GB DDR4 ram with Python version 3.9.18.

The forecasting capability of the proposed model has been tested on different time series. The first group consists of nine-time series TAIEX, SP500_a_ (The dataset SP500_a_ is the daily averages of S&P 500 stock index in [37], while SP500_b_ is the daily open data of S&P 500 in [38]), NASDAQ, Dow Jones, BTC–USD, ETH–USD, EUR–GBP, EUR–USD, and GBP–USD for comparison with existing non-stationary fuzzy time series forecasting models. The second group includes eight classical time series datasets from various domains (Sunspot, MG, SP500_b_, Radio, Lake, CO_2_, Milk, and DJ) for comparison with state-of-the-art batch learning models.

**Figure 2:**
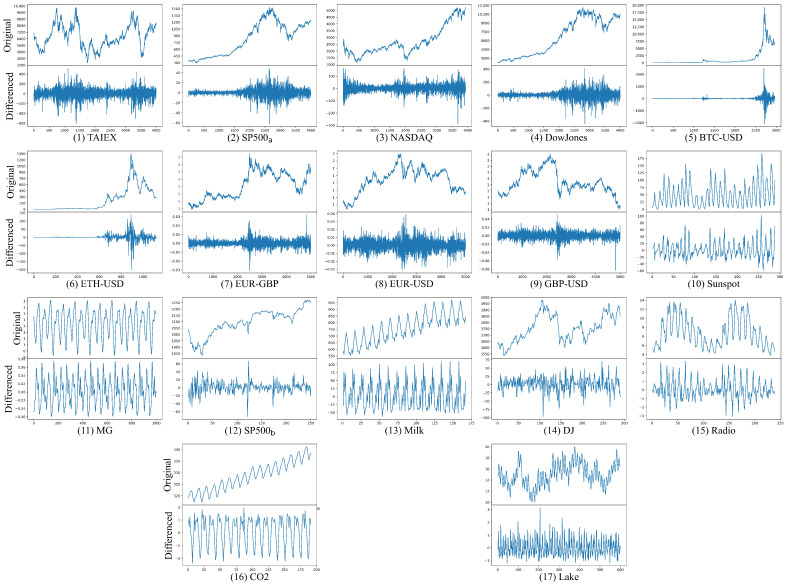
Original and first-order differenced time series for seventeen datasets. The top panel depicts the original time series data. The lower panel shows the first-order differenced time series.

Table 1 summarizes the details of each time series. Figure 2 shows the original and first-order differenced time series for the seventeen-time series. Figure 2 demonstrates that all-time series, excluding MG and CO_2_, display varying trends and heteroscedasticity. While first-order differencing effectively reduces trend non-stationarity in these time series, heteroscedastic characteristics persist. To assess the stationarity properties, we conducted the Augmented Dickey–Fuller (ADF) test and Levene’s test on both original and first-order differenced series. Test results are presented in Table 2. The ADF test was employed to examine the presence of unit roots, with the null hypothesis $H_{0}$ indicating non-stationarity (presence of unit root) and the alternative hypothesis $H_{1}$ suggesting stationarity (absence of unit root) at a significance level α=0.05. Additionally, we applied Levene’s test to evaluate variance homogeneity, where $H_{0}$ represents homoscedasticity (equal variances), and $H_{1}$ indicates heteroscedasticity (unequal variances) at a significance level α=0.05.

The ADF test results reveal that all original time series accept the null hypothesis $H_{0}$ except MG, confirming their non-stationarity. After first-order differencing, all-time series reject $H_{0}$, showing that differencing effectively mitigates non-stationarity. According to Levene’s test results, heteroscedasticity persists in both original and differenced series for all datasets except MG. This analysis shows that while differencing effectively mitigates non-stationarity, variance instability continues to be a significant characteristic in most time series. These findings highlight the complex nature of non-stationarity in time series.

The forecasting performance is quantified using root mean squared error (RMSE), mean absolute percentage error (MAPE), and Theil’s U statistic (U) [25,40,41]. RMSE calculates the divergence between the predicted and actual values. MAPE measures a scale-independent error, allowing direct comparison across datasets. Theil’s U statistic evaluates the forecasting performance of a model compared with the naive method. These metrics are presented as follows:

(18) $$\begin{array}{cc} \; & \mathrm{RMSE}=\sqrt{\frac{1}{N_{te}}\sum_{t=1}^{N_{te}}{\left(y_{t}-{\hat{y}}_{t}\right)}^{2}} \end{array}$$

(19) $$\begin{array}{cc} \; & \mathrm{MAPE}=\frac{1}{N_{te}}\sum_{t=1}^{N_{te}}\left|\frac{y_{t}-{\hat{y}}_{t}}{y_{t}}\right|*100 \end{array}$$

(20) $$\begin{array}{cc} \; & U=\frac{\sqrt{\frac{1}{N_{te}}\sum_{t=1}^{N_{te}}{\left(y_{t}-{\hat{y}}_{t}\right)}^{2}}}{\sqrt{\frac{1}{N_{te}}\sum_{t=1}^{N_{te}}{\left(y_{t}-y_{t-1}\right)}^{2}}}. \end{array}$$

### 4.2. Comparison with Non-Stationary Fuzzy Time Series Forecasting Models

In this section, we conduct comprehensive experiments to evaluate the performance of the proposed TV-NS-BN-PWFTS model against other state-of-the-art non-stationary fuzzy time series forecasting methods. The benchmark FTSFMs include two time-variant FTSFMs [23] (TV-PWFTS, TV-BN-PWFTS) that utilize PWFTS [10] and BN-PWFTS [33] as internal methods, respectively. Incremental ensemble approaches [23] are also applied to construct non-stationary FTSFMs by combining with PWFTS or BN-PWFTS, specifically IE-PWFTS and IE-BN-PWFTS. NSFTS [23] is employed, which maintains constant fuzzy relationships while employing residual-based non-stationary fuzzy sets (Source code for NSFTS, TV-PWFTS, and IE-PWFTS is available on https://github.com/PYFTS/NSFTS (accessed on 31 November 2024). TV-BN-PWFTS, IE-BN-PWFTS, and TV-NS-BN-PWFTS are implemented using the pyFTS library (https://github.com/PYFTS (accessed on 31 November 2024)) for the time-variant framework and pgmpy (https://github.com/pgmpy (accessed on 31 November 2024)) for Bayesian network components). The division of training and testing sets for the first group of datasets is as follows: the first 10% of the data is used for training, and the remaining 90% is used for testing. All experiments were conducted on a Windows 11 desktop computer equipped with an Intel Core i5-13400F processor and 16 GB DDR4 RAM, running Python 3.9.18. We implemented a grid search to identify the optimal parameters for benchmark FTSFMs. For the proposed TV-NS-BN-PWFTS method, we conducted systematic parameter optimization experiments within specified parameter ranges. Parameters are selected from the following ranges: FTSFM order ω {2,3}, number of fuzzy sets from three to fourteen, old data window length $l_{o}$ {100,300}, new data window length $l_{n}$ {50,100}, prediction sub-window length $l_{p}$ {10,30}, model update threshold θ {0.25,1}, and BN adaptive learning weighted parameter η {0.25,0.75}.

Table 3, Table 4 and Table 5 present the comparative prediction performance of six FTSFMs on nine non-stationary time series. Experimental results reveal that the proposed TV-NS-BN-PWFTS achieves superior performance across the majority of datasets. Compared with other BN-PWFTS-based models (IE-BN-PWFTS and TV-BN-PWFTS), the dynamic historical information integration mechanism in TV-NS-BN-PWFTS effectively enhances the FTSFM’s adaptability to the dynamic characteristics of time series. The superior performance of IE-BN-PWFTS over TV-BN-PWFTS further validates the necessity of extracting useful information from historical data for prediction enhancement. Our model’s superior performance over NSFTS reveals that merely adjusting fuzzy set parameters is insufficient to comprehensively capture statistical characteristic changes in time series, reflecting the complex nature of time series non-stationarity. Although TV-NS-BN-PWFTS has slightly higher MAPE and U-values (less than 1.5% difference) compared with IE-BN-PWFTS on the Dow Jones dataset, it retains the optimal RMSE value. This indicates that TV-NS-BN-PWFTS still maintains a highly competitive overall forecasting performance.

**Figure 3:**
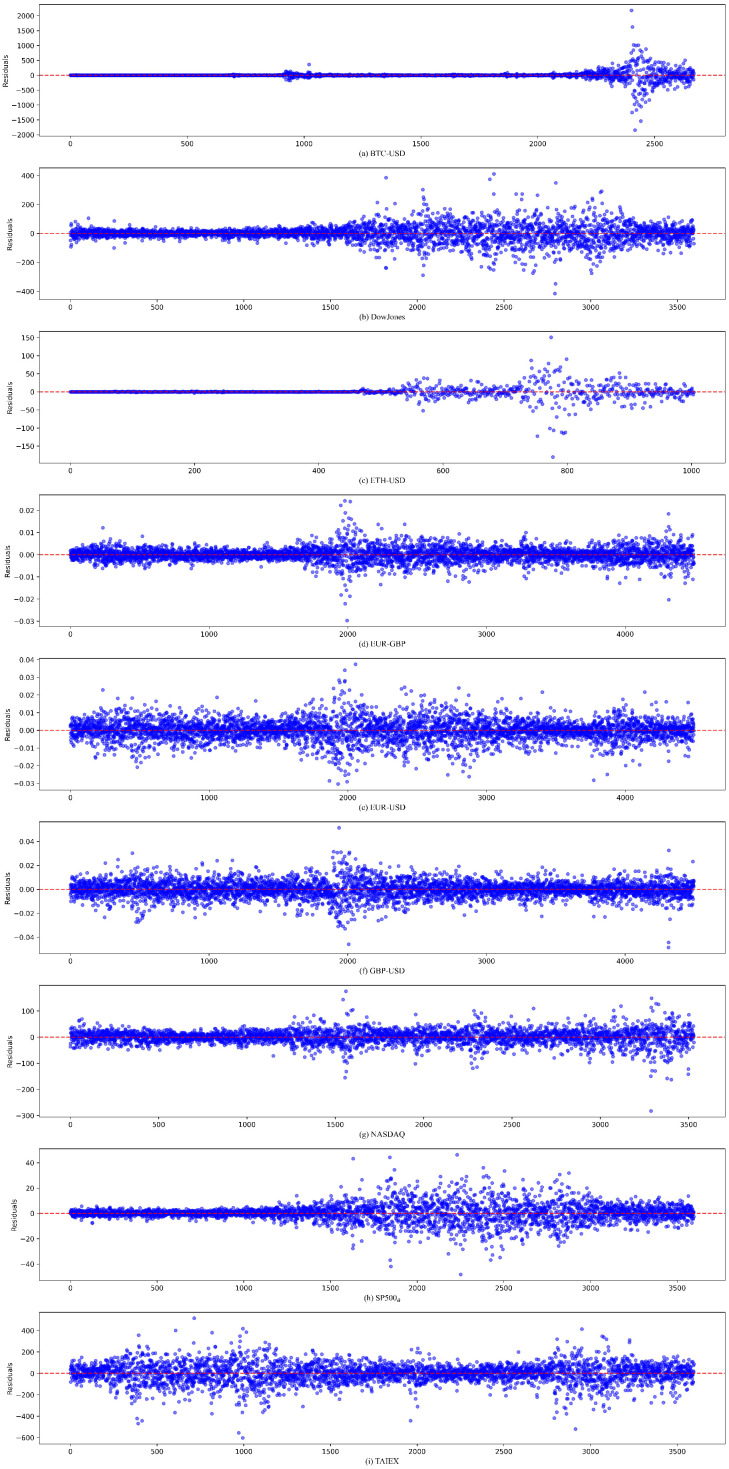
Error scatter plot produced by the proposed model for (**a**) BTC–USD time series, (**b**) Dow Jones time series, (**c**) ETH–USD time series, (**d**) EUR–GBP time series, (**e**) EUR–USD time series, (**f**) GBP–USD time series, (**g**) NASDAQ time series, (**h**) SP500_a_ time series, (**i**) TAIEX time series.

**Figure 4:**
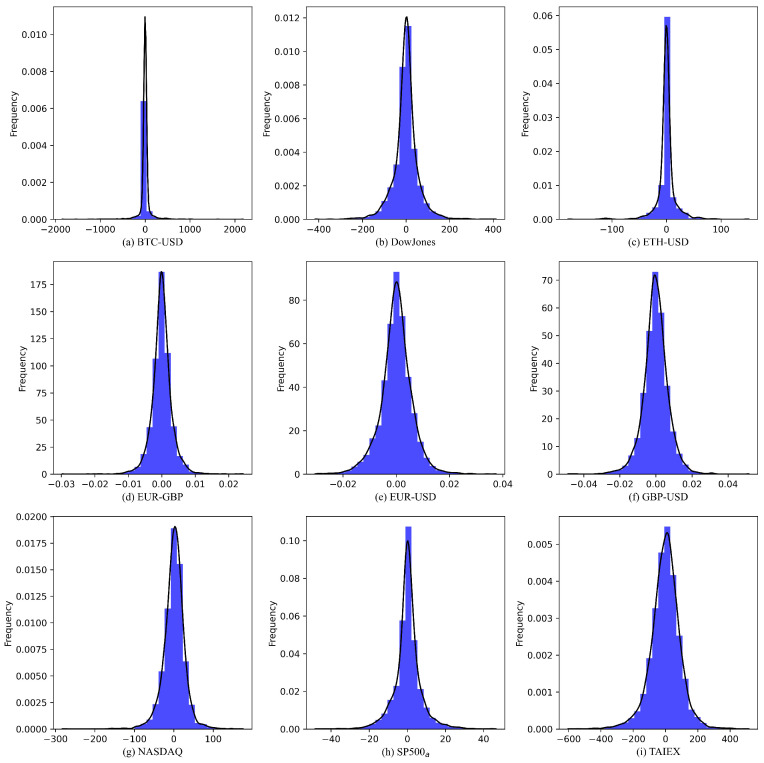
Error distribution histogram produced by the proposed model for (**a**) BTC–USD time series, (**b**) Dow Jones time series, (**c**) ETH–USD time series, (**d**) EUR–GBP time series, (**e**) EUR–USD time series, (**f**) GBP–USD time series, (**g**) NASDAQ time series, (**h**) SP500_a_ time series, (**i**) TAIEX time series.

Figure 3 presents the prediction residuals generated by the proposed model across nine datasets using their respective test subsets. The error values predominantly cluster around zero, indicating minimal overall prediction bias. The absence of periodic patterns or trending behaviors in the residuals suggests that the model effectively captures the dynamic characteristics of the time series. Figure 4 illustrates the residual distribution histograms and their corresponding density curves across the nine-time series. The experimental results demonstrate that the residuals predominantly exhibit characteristics of a standard normal distribution, validating the model’s effectiveness. While minor deviations from standard normality were detected in the EUR–GBP, NASDAQ, TAIEX, and EUR–USD datasets, the model maintains robust reliability and stability overall.

Furthermore, to facilitate a more extensive and thorough evaluation of TV-NS-BN-PWFTS’s forecasting capabilities, we conducted analyses on the TAIEX, NASDAQ, Dow Jones, and SP500 datasets spanning from 2017 to 2022, with each fiscal year treated as an independent time series. The experimental data were sourced from [42]. For comparative analysis purposes, we adopted their experimental configuration where the initial 22-week period constituted the training dataset, and predictions were conducted on a weekly interval basis. The proposed model was benchmarked against several established time-variant fuzzy forecasting models, including NSFTS, the dynamic evolving neural-fuzzy inference system (DENFIS) [43], and the phase-cum-time variant fuzzy time series model (PTVFTS) [42]. The comparative results, presented in Table 6, illustrate the performance metrics in terms of RMSE and MAPE. The empirical evidence clearly indicates that the proposed TV-NS-BN-PWFTS demonstrates markedly superior predictive performance when benchmarked against other contemporary fuzzy forecasting models. Table 6 demonstrates the significant advantages of TV-NS-BN-PWFTS. The model achieved optimal performance in both RMSE and MAPE metrics across sixteen out of twenty-four annual prediction tasks. While PTVFTS outperformed TV-NS-BN-PWFTS in six specific years (SP500-2021, TAIEX-2017, NASDAQ-2018, SP500-2018, NASDAQ-2021, and Dow Jones-2018), TV-NS-BN-PWFTS maintained consistently high prediction accuracy across the majority of forecasting tasks. The model demonstrated an average performance improvement of over 30.50% across sixteen datasets. In the remaining six datasets where PTVFTS showed superior results, TV-NS-BN-PWFTS exhibited relatively minor performance gaps of 20.16% in RMSE and 24.58% in MAPE metrics.

**Figure 5:**
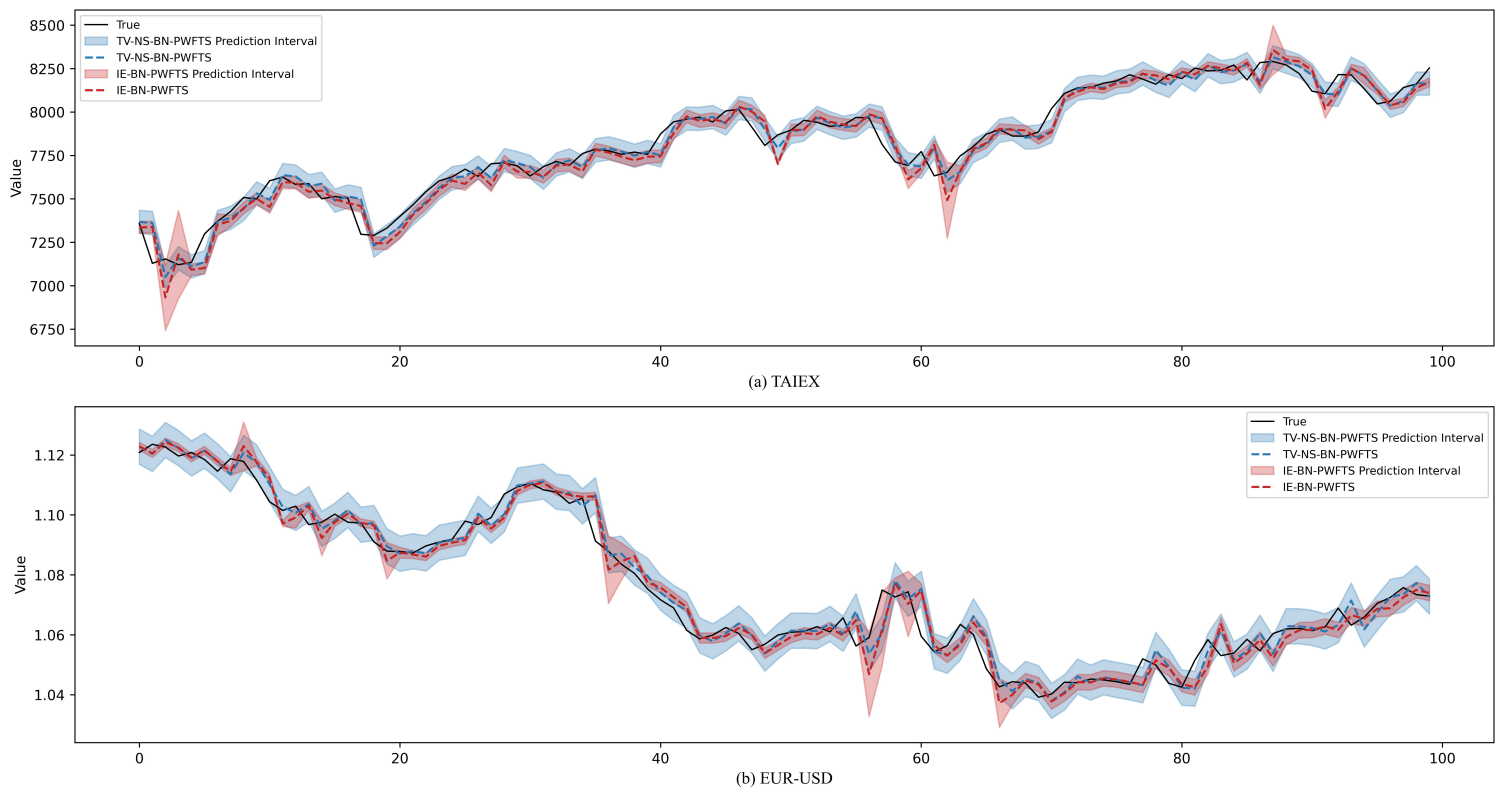
Prediction intervals yielded by the proposed model and IE-BN-PWFTS for (**a**) TAIEX time series and (**b**) EUR–USD time series.

To evaluate the capability of TV-NS-BN-PWFTS in quantifying prediction uncertainty, we extended TV-NS-BN-PWFTS to construct prediction intervals. Specifically, TV-NS-BN-PWFTS adopts the interval prediction methodology proposed by [10], utilizing fuzzy empirical probability-weighted FLRGs to compute prediction intervals. Two representative non-stationary financial time series, TAIEX and EUR–USD, were selected for experimental analysis. For clarity of presentation, we conducted a detailed analysis of the last 100 observations in the test set. As illustrated in Figure 5, the prediction intervals generated by TV-NS-BN-PWFTS achieved higher true value coverage rates compared with IE-BN-PWFTS, validating the superiority and reliability of our proposed model in non-stationary time series forecasting.

### 4.3. Comparison with Batch Learning Models

In this section, the proposed model is compared with various outstanding batch learners in the second dataset group to evaluate its predictive performance. We compared the proposed method with classic predictive models such as the multiresolution autoregressive model (MAR), the autoregressive model (AR), the adaptive network-based fuzzy inference system (ANFIS), and the artificial neural network (ANN). Additionally, deep neural network-based models, such as temporal convolutional networks (TCNs), recurrent neural networks (RNNs), the long short-term memory (LSTM) network, and gated recurrent unit (GRU), are included in the comparison. The fuzzy cognitive map (FCM) models integrated with wavelet transform (Wavelet-HFCM [39]) or convolutional neural network (CNN-FCM [44]) are also included in the comparison. Wavelet-HFCM utilizes the redundant wavelet transform to decompose non-stationary series into multivariate time series. HFCM models the latent relationships within these time series and predicts these time series. CNN-FCM applies FCM to learn the relationships between series decomposed by TCN. A regression model then predicts the next observation based on the FCM output. The fuzzy-probabilistic predictive models PWFTS [10] and BN-PWFTS [33] are also employed. This experiment adopts the dataset division scheme in [44]. Considering the characteristics of the second dataset group, the search range for the model order is set from three to thirteen to achieve optimal predictive performance. The optimal results for PWFTS and BN-PWFTS were determined using the grid search method. The results for other benchmark methods were obtained from [39,44].

Table 7 indicates that TV-NS-BN-PWFTS achieves the best forecasting performance on four out of eight datasets while maintaining competitive performance just behind BN-PWFTS on CO_2_, Lake, and Milk datasets. Compared with BN-PWFTS, TV-NS-BN-PWFTS exhibits stronger adaptability to dynamic changes in non-stationary time series due to its model update strategy driven by phased prediction performance. For datasets like DJ, where significant differences exist between training and testing data distributions, the model updating mechanism of TV-NS-BN-PWFTS has notable advantages. The dynamic adjustment mechanism may introduce extra fluctuations when the time series has strong periodic characteristics, such as CO_2_ and Milk datasets. TV-NS-BN-PWFTS surpasses FCM-based methods on seven out of eight datasets except for MG. The superior performance over Wavelet-HFCM and CNN-FCM can be attributed to its ability to capture the dynamic changes in temporal patterns of non-stationary time series, which FCM methods with fixed causal relationships cannot achieve. TV-NS-BN-PWFTS’s weaker performance on the MG dataset may be due to the absence of non-stationarity, which prevents the update mechanism of TV-NS-BN-PWFTS from fully exhibiting its advantages. This indirectly proves that the model is more suitable for handling complex time series with non-stationary characteristics.

**Figure 6:**
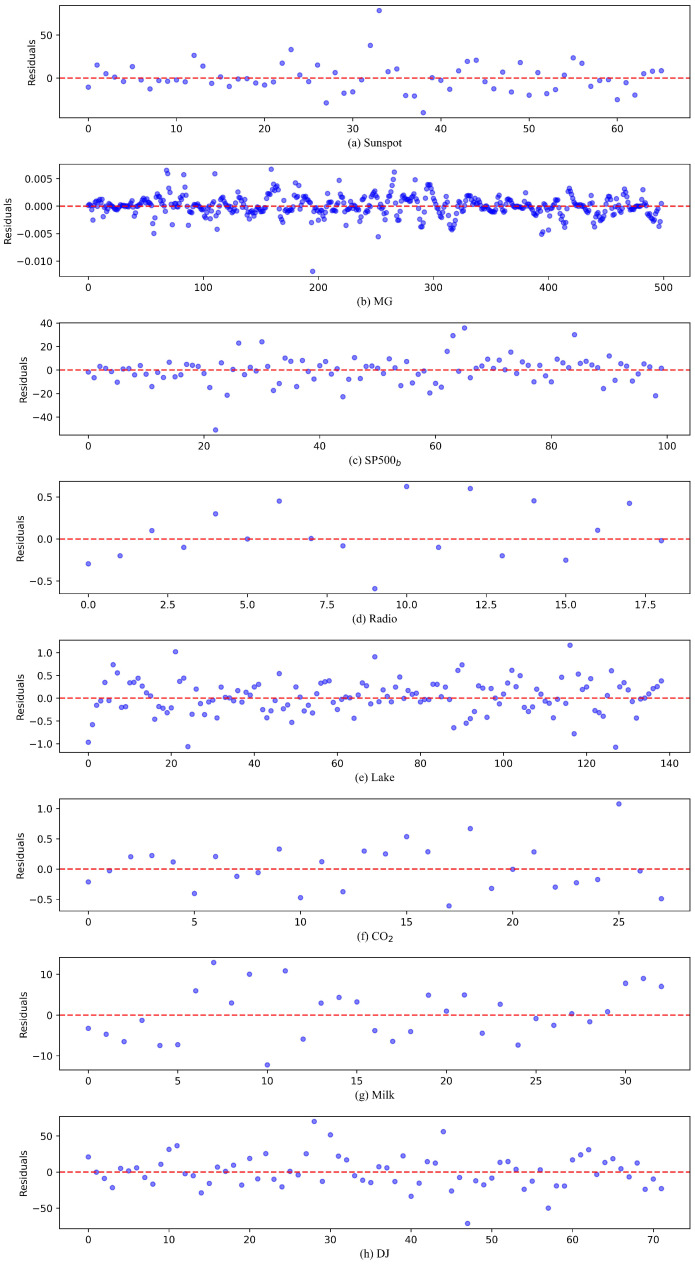
Error scatter plot produced by the proposed model for (**a**) Sunspot time series, (**b**) MG time series, (**c**) SP500_b_ time series, (**d**) Radio time series, (**e**) Lake time series, (**f**) CO_2_ time series, (**g**) Milk time series, (**h**) DJ time series.

**Figure 7:**
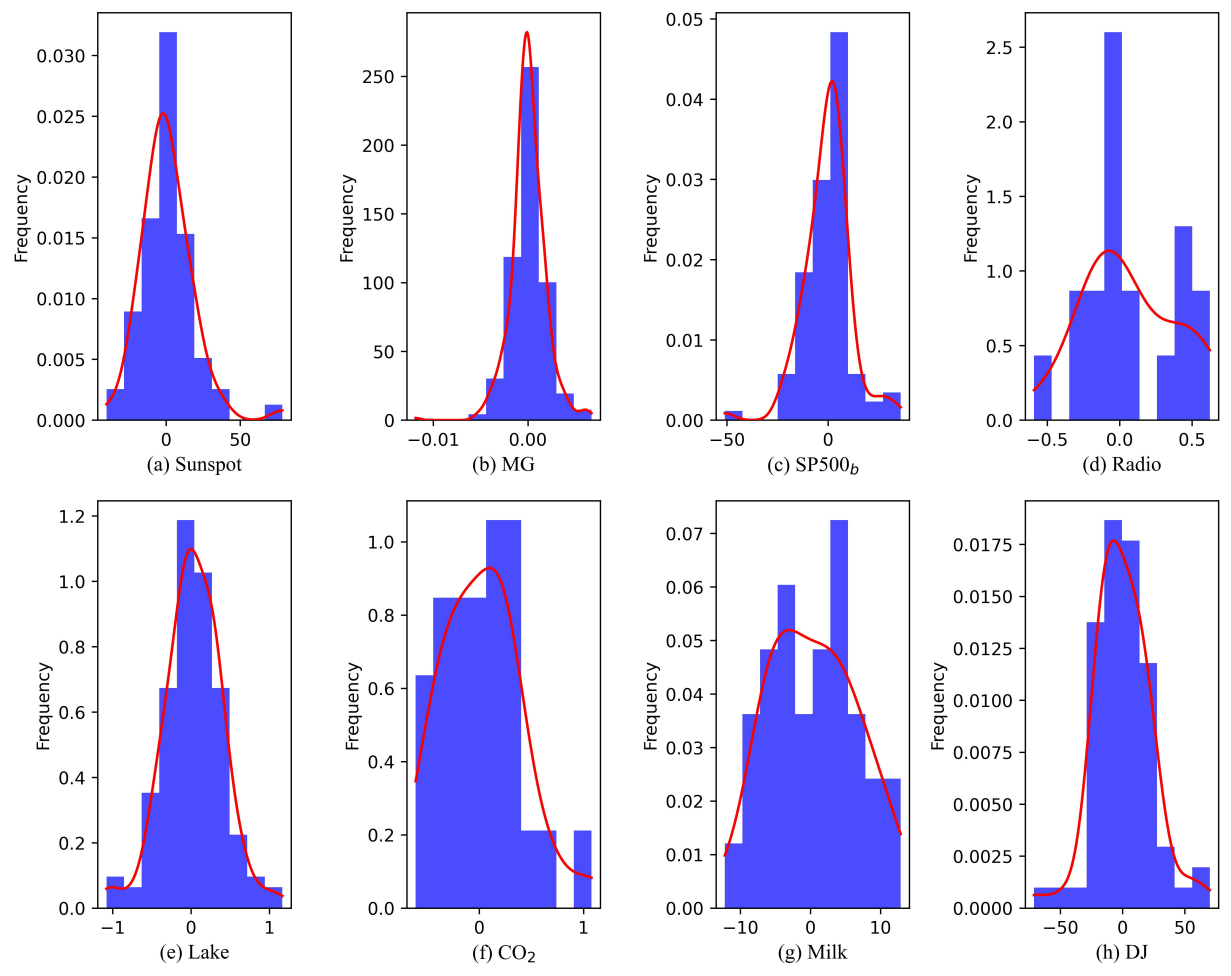
Error distribution histogram produced by the proposed model for (**a**) Sunspot time series, (**b**) MG time series, (**c**) SP500_b_ time series, (**d**) Radio time series, (**e**) Lake time series, (**f**) CO_2_ time series, (**g**) Milk time series, (**h**) DJ time series.

Figure 6 demonstrates the prediction residuals obtained from the proposed model for eight datasets when benchmarked against batch learning approaches. The balanced distribution of positive and negative errors reveals the model’s ability to generate unbiased predictions without systematic overestimation or underestimation. The absence of temporal patterns in the residual scatter plots confirms the model’s effectiveness in capturing time-varying characteristics of the data. Figure 7 presents the residual distribution characteristics across eight datasets. The SP500_b_, DJ, and Lake datasets exhibit highly symmetric normal distributions. The density curves for the Sunspot, CO_2_, and MG datasets display varying degrees of skewness, while the Milk and Radio datasets demonstrate bimodal distribution patterns, potentially attributable to limited sample sizes and inherent data fluctuations.

To further validate the performance of the proposed model, we conducted comparative experiments using NASDAQ daily closing prices from 2001 to 2012 [13]. The model was benchmarked against various FTSFMs with data stationary operations and classical time series forecasting models. The comparison methods include an FTSFM with an improved sparrow search algorithm and complete ensemble empirical mode decomposition with adaptive noises (CEEMDAN-ISSA-FTS) [13], the FTSFM based on fuzzy c-means clustering and the empirical mode decomposition method (EMD-FC-FTS) [45] utilizing empirical mode decomposition, Wavelet-HFCM [39] employing wavelet transform, prophet [46] based on time series additive decomposition, and traditional Chen’s FTSFM (Chen) [6] and ARIMA using the differencing method. As shown in Table 8, TV-NS-BN-PWFTS outperforms these methods in both RMSE and MAPE metrics, demonstrating its significant advantages over traditional feature extraction-based time series prediction models and further validating its effectiveness in handling non-stationary time series forecasting tasks.

### 4.4. Comparison Considering Multiple Time Series Together

To comprehensively evaluate model performance across all datasets, we conducted the Friedman test [47] and the post-hoc Holm test [48] for non-parametric statistical analysis. The results in Table 9 display TV-NS-BN-PWFTS’s superior performance with an average ranking of 1.06 across nine datasets, considerably outperforming other non-stationary FTSFMs. The Friedman test results include a test statistic *z*-value of 42.7070 and a *p*-value of 4.2364 × 10^−8^, indicating significant performance differences among the FTSFMs at the 5% significance level.

The Holm test results in Table 10 further reveal that TV-NS-BN-PWFTS has statistically significant performance advantages over IE-PWFTS, TV-BN-PWFTS, TV-PWFTS, and NSFTS. In contrast, the performance comparison between TV-NS-BN-PWFTS and IE-BN-PWFTS shows no statistically significant difference.

Statistical comparison with batch learning models through Friedman and Holm tests demonstrates TV-NS-BN-PWFTS’s effectiveness. According to Table 11, TV-NS-BN-PWFTS achieves the top average rank of 2.25 compared with twelve batch learners, notably excelling in predicting time series with complex dynamic characteristics. Friedman test results, displaying a test statistic *z*-value of 47.7997 and a *p*-value of 3.3867 × 10^−6^, reveal significant performance differences among models at the 5% significance level. The Holm test in Table 12 confirms the statistically significant difference of TV-NS-BN-PWFTS over AR, TCN, ANN, GRU, MAR, LSTM, and ANFIS at the 95% confidence level. Despite showing non-significant differences with RNN, CNN-FCM, PWFTS, Wavelet-HFCM, and BN-PWFTS, TV-NS-BN-PWFTS still demonstrates superior overall predictive accuracy according to the RMSE metric.

### 4.5. Ablation Study

In this section, we conduct ablation experiments to validate the effectiveness of core modules. We first introduce three variants of TV-NS-BN-PWFTS. TV-BN-PWFTS represents an FTSFM that employs BN-PWFTS as its core module with a traditional time-variant updating strategy [15]. This variant only utilizes recent data to construct a new BN-PWFTS for prediction, disregarding useful historical information. TV-BN-PWFTS (NSFS) replaces traditional fuzzy sets with our proposed non-stationary fuzzy sets to demonstrate their effectiveness. TV-BN-PWFTS (adaptive) substitutes the time-variant updating strategy [15] with our dynamic updating approach to verify its efficacy in capturing temporal patterns. Table 13 presents the results across nine time series using three metrics: RMSE, MAPE, and U. The results show that both variant methods incorporating either non-stationary fuzzy sets or model updating modules outperform the baseline TV-BN-PWFTS. The superior performance of TV-BN-PWFTS (adaptive) over TV-BN-PWFTS (NSFS) indicates that dynamic changes in temporal patterns significantly impact the long-term prediction of non-stationary time series. TV-NS-BN-PWFTS offers a comprehensive approach to handling non-stationarity in time series. It not only addresses the dynamic changes in vagueness through non-stationary fuzzy sets but also captures the evolution of temporal relationships, thereby providing more accurate and comprehensive forecasting results.

## 5. Conclusions

To address the negative impact of non-stationarity on FTSFM forecasting performance, we propose a novel hybrid FTSFM that effectively captures heteroscedasticity and trend changes inherent in time series. It employs a novel dynamic updating scheme that effectively incorporates historical information with new data. First-order differencing reduces time series non-stationarity while extracting time series variation information. Dynamic adjustment of non-stationary fuzzy set parameters based on residuals enables precise modeling of local changes in time series variation. Once old and new data are fuzzified, the model rebuilds fuzzy empirical probability-based FLRGs, enabling dynamic updates of fuzzy relationships. We use adaptive BN structure learning to model dependence relationships dynamically between time points in FLRGs. The updates of the BN and FLRGs reflect changes in temporal relationships within the time series. The proposed hybrid FTSFM successfully integrates historical knowledge preservation with dynamic adaptation according to new data, enhancing FTSFMs’ ability to handle non-stationary time series more effectively. Experimental results show that the proposed model outperforms existing non-stationary FTSFMs and batch learning models. Hypothesis tests verify the reliability of the proposed model.

The proposed model demonstrates excellent performance in improving the forecasting accuracy of FTSFM for non-stationary time series. However, several issues need further investigation. While the current model successfully handles univariate non-stationary time series forecasting, future work will extend it to address the challenges of multivariate non-stationary time series prediction. The current research is limited to using triangular membership functions to construct non-stationary fuzzy sets. In order to expand the applicability of the model, it is necessary to conduct in-depth research on the performance of other types of membership functions, such as Gaussian functions and ladder functions, in non-stationary environments. Due to time series non-stationarity, quantifying prediction uncertainty is essential. We plan to incorporate confidence interval estimation into our forecasting framework to better assess prediction reliability. The potential integration of fuzzy reasoning and neural network fitting will be explored, which may combine the continuous-time modeling advantages of NARX while preserving the interpretability characteristics of fuzzy systems.

## Figures and Tables

**Table 1:** Descriptions of seventeen time series.

Dataset	Description	Number
TAIEX [37]	Daily averages of open, high, low, and close prices for the Dow Jones Industrial Average	4000
SP500_a_ [37]	Daily averages of open, high, low, and close prices for the S&P 500 stock index	4000
NASDAQ [37]	Daily averages of open, high, low, and close prices for the National Association of Securities Dealers Automated Quotations Composite Index	4000
Dow Jones [37]	Daily averages of the Dow Jones Industrial Index’s open, high, low, and close prices	4000
BTC–USD [37]	Daily cryptocurrency exchange rates for Bitcoin quoted in US Dollars	2968
ETH–USD [37]	Daily cryptocurrency exchange rates for Ethereum quoted in US Dollars	1121
EUR–GBP [37]	FOREX data, including daily average quotations for Euro to Great British Pound	5000
EUR–USD [37]	FOREX data, including daily average quotations for US Dollar to Euro	5000
GBP–USD [37]	FOREX data, including daily average quotations for Great British Pound to US Dollar	5000
Sunspot [38]	Yearly sunspot count	288
MG [38]	Obtained by solving a first-order nonlinear differential-delay equation via the fourth-order Runge–Kutta algorithm	1000
SP500_b_ [38]	Daily open prices of the S&P 500 stock index	251
Milk [38]	Milk production in pounds on a monthly basis	168
DJ [38]	Monthly close prices for the Dow Jones industrial index	291
Radio [38]	Highest permitted radio frequency for broadcasting in Washington, DC, USA	240
CO_2_ [38]	CO_2_ measurements at Mauna Loa	192
Lake [38]	Monthly level of Lake Erie	680

**Table 2:** Stationarity evaluation based on Augmented Dickey–Fuller test and Levene’s test.

Dataset	Original Time Series				First Order Differenced Time Series			
	ADF Test *p* Value	Test Result	Levene’s Test *p* Value	Test Result	ADF Test *p* Value	Test Result	Levene’s Test *p* Value	Test Result
TAIEX	0.1175	$H_{0}$ Accepted	0.0000	$H_{0}$ Rejected	0.0000	$H_{0}$ Rejected	0.0000	$H_{0}$ Rejected
SP500	0.7733	$H_{0}$ Accepted	0.0000	$H_{0}$ Rejected	0.0000	$H_{0}$ Rejected	0.0000	$H_{0}$ Rejected
NASDAQ	0.9841	$H_{0}$ Accepted	0.0000	$H_{0}$ Rejected	0.0000	$H_{0}$ Rejected	0.0000	$H_{0}$ Rejected
Dow Jones	0.8189	$H_{0}$ Accepted	0.0000	$H_{0}$ Rejected	0.0000	$H_{0}$ Rejected	0.0000	$H_{0}$ Rejected
BTC–USD	0.6710	$H_{0}$ Accepted	0.0000	$H_{0}$ Rejected	0.0000	$H_{0}$ Rejected	0.0000	$H_{0}$ Rejected
ETH–USD	0.3546	$H_{0}$ Accepted	0.0000	$H_{0}$ Rejected	0.0000	$H_{0}$ Rejected	0.0000	$H_{0}$ Rejected
EUR–GBP	0.4537	$H_{0}$ Accepted	0.0000	$H_{0}$ Rejected	0.0000	$H_{0}$ Rejected	0.0000	$H_{0}$ Rejected
EUR–USD	0.3579	$H_{0}$ Accepted	0.0000	$H_{0}$ Rejected	0.0000	$H_{0}$ Rejected	0.0000	$H_{0}$ Rejected
GBP–USD	0.7022	$H_{0}$ Accepted	0.0000	$H_{0}$ Rejected	0.0000	$H_{0}$ Rejected	0.0000	$H_{0}$ Rejected
Sunspot	0.1462	$H_{0}$ Accepted	0.0000	$H_{0}$ Rejected	0.0000	$H_{0}$ Rejected	0.0000	$H_{0}$ Rejected
MG	0.0000	$H_{0}$ Rejected	0.9164	$H_{0}$ Accepted	0.0000	$H_{0}$ Rejected	0.9018	$H_{0}$ Accepted
SP500	0.8298	$H_{0}$ Accepted	0.0000	$H_{0}$ Rejected	0.0000	$H_{0}$ Rejected	0.0000	$H_{0}$ Rejected
Milk	0.6274	$H_{0}$ Accepted	0.0102	$H_{0}$ Rejected	0.0301	$H_{0}$ Rejected	0.0110	$H_{0}$ Rejected
DJ	0.3550	$H_{0}$ Accepted	0.0023	$H_{0}$ Rejected	0.0000	$H_{0}$ Rejected	0.0028	$H_{0}$ Rejected
Radio	0.2491	$H_{0}$ Accepted	0.0001	$H_{0}$ Rejected	0.0102	$H_{0}$ Rejected	0.0000	$H_{0}$ Rejected
CO_2_	0.9964	$H_{0}$ Accepted	0.0000	$H_{0}$ Rejected	0.0001	$H_{0}$ Rejected	0.0000	$H_{0}$ Rejected
Lake	0.1109	$H_{0}$ Accepted	0.0135	$H_{0}$ Rejected	0.0000	$H_{0}$ Rejected	0.0098	$H_{0}$ Rejected

**Table 3:** Comparison of the proposed method with other non-stationary fuzzy time series forecasting models in terms of root mean squared error (RMSE). The optimal value is represented in bold.

Dataset	TV-PWFTS	IE-PWFTS	IE-BN-PWFTS	TV-BN-PWFTS	NSFTS	TV-NS-BN-PWFTS
TAIEX	123.9999	1018.5415	95.2133	137.1233	107.4994	**92.9266**
SP500_a_	8.8415	42.5027	7.2578	13.2580	7.8307	**7.1490**
NASDAQ	35.0900	202.5477	28.0960	43.5705	33.7277	**27.5051**
Dow Jones	69.5462	284.9341	57.9956	104.4958	62.6613	**57.7796**
BTC–USD	306.1626	1364.0944	142.1741	197.9182	151.4576	**138.6654**
ETH–USD	44.5400	158.8328	18.8919	27.8365	19.3987	**18.3194**
EUR–USD	0.0069	0.0190	0.0061	0.0117	0.0064	**0.0060**
EUR–GBP	0.0035	0.0048	**0.0031**	0.0061	0.0032	**0.0031**
GBP–USD	0.0083	0.0283	0.0072	0.0141	0.0092	**0.0070**

**Table 4:** Comparison of the proposed method with other non-stationary fuzzy time series forecasting models in terms of mean absolute percentage error. The optimal value is represented in bold.

Dataset	TV-PWFTS	IE-PWFTS	IE-BN-PWFTS	TV-BN-PWFTS	NSFTS	TV-NS-BN-PWFTS
TAIEX	1.4122	10.7081	1.0428	1.5246	1.2096	**1.0174**
SP500_a_	0.6130	1.9956	0.4914	0.9442	0.5505	**0.4881**
NASDAQ	0.9000	4.0233	0.7558	1.1891	0.9791	**0.7534**
Dow Jones	0.6054	1.6009	**0.5049**	0.9514	0.5755	0.5120
BTC–USD	6.5868	36.2601	**2.4370**	3.7092	3.0203	2.5151
ETH–USD	7.3500	37.0502	3.4305	4.8608	3.8407	**3.4196**
EUR–USD	0.3892	0.9249	0.3425	0.6631	0.3642	**0.3402**
EUR–GBP	0.3122	0.3230	0.2725	0.5499	0.2798	**0.2696**
GBP–USD	0.3630	0.8986	0.3163	0.6325	0.3881	**0.3114**

**Table 5:** Comparison of the proposed method with other non-stationary fuzzy time series forecasting models in terms of Theil’s U statistic. The optimal value is represented in bold.

Dataset	TV-PWFTS	IE-PWFTS	IE-BN-PWFTS	TV-BN-PWFTS	NSFTS	TV-NS-BN-PWFTS
TAIEX	1.3047	10.7687	1.0016	1.4497	1.1420	**0.9870**
SP500_a_	1.1164	5.4013	0.9164	1.6742	1.0019	**0.9144**
NASDAQ	1.2500	7.2834	1.0102	1.5615	1.2154	**0.9913**
Dow Jones	1.1043	4.5522	**0.9207**	1.6689	1.0062	0.9275
BTC–USD	1.9831	8.9213	0.9205	1.2916	0.9999	**0.9151**
ETH–USD	2.0600	7.9795	0.9487	1.3920	1.0003	**0.9432**
EUR–USD	1.1296	3.1134	0.9902	1.9032	1.0476	**0.9845**
EUR–GBP	1.1054	1.4879	**0.9669**	1.8933	1.0055	0.9745
GBP–USD	1.1247	3.8410	0.9789	1.9140	1.2580	**0.9581**

**Table 6:** Prediction performance of the models on NASDAQ, SP500, Dow Jones, and TAIEX 2017–2022 in terms of RMSE and MAPE. The optimal value is represented in bold.

Dataset	RMSE				MAPE			
	NSFTS	DENFIS	PTVFTS	TV-NS-BN-PWFTS	NSFTS	DENFIS	PTVFTS	TV-NS-BN-PWFTS
NASDAQ-2017	108.7284	239.1259	48.1924	**36.8277**	1.3742	2.9002	0.5577	**0.4102**
NASDAQ-2018	176.4645	632.8676	**115.4398**	136.8919	1.8796	6.9452	**1.1441**	1.5356
NASDAQ-2019	142.2725	443.8779	81.5571	**42.4718**	1.4696	4.2853	0.7909	**0.3875**
NASDAQ-2020	268.2565	869.2766	199.9467	**96.4991**	1.9631	5.9934	1.4795	**0.5843**
NASDAQ-2021	295.9192	679.2459	**159.6753**	199.3805	1.5915	3.6026	**0.8272**	1.0269
NASDAQ-2022	284.4377	481.7911	238.0055	**204.9993**	2.1808	3.5995	1.6000	**1.3231**
SP500-2017	24.8333	78.8404	13.0605	**9.9889**	0.7286	2.4440	0.3908	**0.2895**
SP500-2018	51.3791	187.3747	**31.8340**	41.7365	1.5853	5.4565	**0.8731**	1.2067
SP500-2019	52.9810	125.7938	24.2265	**12.6155**	1.5685	3.3177	0.6088	**0.3087**
SP500-2020	74.2154	202.9605	60.1607	**24.4032**	1.8372	4.7154	1.4894	**0.5327**
SP500-2021	87.8825	190.3767	**40.0989**	44.8478	1.6162	3.3508	**0.7340**	0.7523
SP500-2022	83.9869	185.1545	64.8664	**58.3521**	1.7117	3.9391	1.2873	**1.0746**
Dow Jones-2017	155.6373	1099.0488	105.9790	**101.4949**	0.4887	3.7595	0.3769	**0.3240**
Dow Jones-2018	460.0347	1473.7939	**300.8507**	402.6199	1.4994	4.5950	**0.8755**	1.2875
Dow Jones-2019	512.6081	632.8676	234.7223	**125.2802**	1.7099	6.9452	0.6574	**0.3313**
Dow Jones-2020	606.3734	885.0938	515.7433	**249.5781**	1.7313	2.6101	1.5478	**0.6366**
Dow Jones-2021	610.8571	1023.9746	**283.7141**	308.0270	1.5315	2.3345	0.6558	**0.6414**
Dow Jones-2022	587.7988	2435.7356	445.4243	**393.2805**	1.4833	6.1544	1.1168	**0.8883**
TAIEX-2017	172.0256	218.0463	**64.5821**	65.0468	1.4130	1.6605	**0.4914**	0.4982
TAIEX-2018	163.9537	623.0989	109.0077	**91.3104**	1.1107	4.6290	**0.7166**	0.7262
TAIEX-2019	179.5368	514.1494	77.9204	**67.5768**	1.3398	3.6155	0.5358	**0.4666**
TAIEX-2020	218.2937	961.8291	145.8409	**116.1398**	1.3034	5.7339	0.9152	**0.6687**
TAIEX-2021	221.4625	679.2459	159.8782	**100.8645**	1.0444	3.6026	0.7338	**0.4578**
TAIEX-2022	323.6182	1006.9976	233.6213	**174.7879**	1.8401	5.8642	1.2132	**0.9504**

**Table 7:** Comparison of the proposed method with batch learning models in terms of RMSE. The optimal value is represented in bold.

	CO_2_	DJ	Lake	MG	Milk	Radio	SP500_b_	Sunspot
RNN	1.4190	26.2320	0.3740	**0.0010**	29.2530	0.6130	27.8960	19.2920
ANFIS	0.9100	27.5260	0.4580	**0.0010**	9.5780	0.6510	14.9350	22.7530
LSTM	2.1600	26.9360	0.3840	**0.0010**	32.7430	0.5900	46.2660	19.0060
ANN	1.6950	28.5320	0.4020	0.0050	27.1130	0.6520	17.6960	19.9010
AR	1.3500	29.8220	0.6380	0.0350	57.7170	0.9020	17.8970	35.2620
MAR	0.8120	26.7330	0.3900	0.0020	37.8380	0.6620	16.0410	19.1860
GRU	1.5610	25.2110	0.3850	**0.0010**	36.0940	0.8320	20.4070	19.4080
TCN	3.1200	25.2140	0.4090	**0.0010**	33.8580	0.6020	51.2670	22.4490
Wavelet-HFCM	0.5600	23.1590	0.3770	0.0040	8.2580	0.5470	16.1050	18.9160
CNN-FCM	0.7310	25.1900	0.3910	**0.0010**	30.4740	0.5670	20.8160	17.9490
PWFTS	0.4884	22.6454	0.3816	0.0050	8.3004	0.3705	11.6922	23.6950
BN-PWFTS	**0.3412**	22.9275	**0.3663**	0.0013	**6.0392**	0.3290	11.7978	18.8784
TV-NS-BN-PWFTS	0.3757	**22.5617**	0.3692	0.0018	6.1244	**0.3289**	**11.5956**	**17.5088**

**Table 8:** Results of the proposed model on NASDAQ 2001-2012 in terms of RMSE and MAPE. The optimal value is represented in bold.

	CEEMDAN-ISSA-FTS	Chen	ARIMA	Prophet	EMD-FC-FTS	Wavelet-HFCM	TV-NS-BN-PWFTS
RMSE	45.8600	80.4000	97.6200	120.0600	126.9500	74.4300	**29.3285**
MAPE	1.2200	2.3300	2.7300	3.4800	3.4700	2.1200	**0.7574**

**Table 9:** Rankings of non-stationary fuzzy time series forecasting models across nine datasets. The optimal value is represented in bold.

Dataset	TV-PWFTS	IE-PWFTS	IE-BN-PWFTS	TV-BN-PWFTS	NSFTS	TV-NS-BN-PWFTS
TAIEX	4.00	6.00	2.00	5.00	3.00	**1.00**
SP500	4.00	6.00	2.00	5.00	3.00	**1.00**
NASDAQ	4.00	6.00	2.00	5.00	3.00	**1.00**
Dow Jones	4.00	6.00	2.00	5.00	3.00	**1.00**
BTC–USD	5.00	6.00	2.00	4.00	3.00	**1.00**
ETH–USD	5.00	6.00	2.00	4.00	3.00	**1.00**
EUR–USD	4.00	6.00	2.00	5.00	3.00	**1.00**
EUR–GBP	4.00	5.00	**1.50**	6.00	3.00	**1.50**
GBP–USD	3.00	6.00	2.00	5.00	4.00	**1.00**
Average	4.11	5.89	1.94	4.89	3.11	**1.06**

**Table 10:** Holm test results of non-stationary fuzzy time series forecasting models.

	Comparison	*z*-Value	*p*-Value
1	TV-NS-BN-PWFTS vs. IE-PWFTS	5.4805	0.0000
2	TV-NS-BN-PWFTS vs. TV-BN-PWFTS	4.3466	0.0000
3	TV-NS-BN-PWFTS vs. TV-PWFTS	3.4647	0.0027
4	TV-NS-BN-PWFTS vs. NSFTS	2.3308	0.0198
5	TV-NS-BN-PWFTS vs. IE-BN-PWFTS	1.0079	0.9405

**Table 11:** Rankings of the proposed method and batch learning models across eight datasets. The optimal value is represented in bold.

Methods	CO_2_	DJ	Lake	MG	Milk	Radio	SP500_b_	Sunspot	Average
RNN	9.00	8.00	3.00	**3.50**	7.00	8.00	11.00	7.00	7.06
ANFIS	7.00	11.00	12.00	**3.50**	5.00	9.00	4.00	11.00	7.81
LSTM	12.00	10.00	6.00	**3.50**	9.00	6.00	12.00	5.00	7.94
ANN	11.00	12.00	10.00	11.50	6.00	10.00	7.00	9.00	9.56
AR	8.00	13.00	13.00	13.00	13.00	13.00	8.00	13.00	11.75
MAR	6.00	9.00	8.00	9.00	12.00	11.00	5.00	6.00	8.25
GRU	10.00	6.00	7.00	**3.50**	11.00	12.00	9.00	8.00	8.31
TCN	13.00	7.00	11.00	**3.50**	10.00	7.00	13.00	10.00	9.31
Wavelet-HFCM	4.00	4.00	4.00	10.00	3.00	4.00	6.00	4.00	4.88
CNN-FCM	5.00	5.00	9.00	**3.50**	8.00	5.00	10.00	2.00	5.94
PWFTS	3.00	2.00	5.00	11.50	4.00	3.00	2.00	12.00	5.31
BN-PWFTS	**1.00**	3.00	**1.00**	7.00	**1.00**	2.00	3.00	3.00	2.62
TV-NS-BN-PWFTS	2.00	**1.00**	2.00	8.00	2.00	**1.00**	**1.00**	**1.00**	**2.25**

**Table 12:** Holm test results of the proposed method and batch learning models.

	Comparison	*z*-Value	*p*-Value
1	TV-NS-BN-PWFTS vs. AR	4.8787	0.0000
2	TV-NS-BN-PWFTS vs. TCN	3.6270	0.0014
3	TV-NS-BN-PWFTS vs. ANN	3.7554	0.0016
4	TV-NS-BN-PWFTS vs. GRU	3.1134	0.0111
5	TV-NS-BN-PWFTS vs. MAR	3.0813	0.0144
6	TV-NS-BN-PWFTS vs. LSTM	2.9208	0.0349
7	TV-NS-BN-PWFTS vs. ANFIS	2.8566	0.0471
8	TV-NS-BN-PWFTS vs. RNN	2.4715	0.1615
9	TV-NS-BN-PWFTS vs. CNN-FCM	1.8937	0.1748
10	TV-NS-BN-PWFTS vs. PWFTS	1.5728	0.2316
11	TV-NS-BN-PWFTS vs. Wavelet-HFCM	1.3481	0.7105
12	TV-NS-BN-PWFTS vs. BN-PWFTS	0.1926	0.8473

**Table 13:** Ablation study in terms of RMSE, MAPE, and U.

		TAIEX	SP500	NASDAQ	Dow Jones	BTC–USD	ETH–USD	EUR–USD	EUR–GBP	GBP–USD
RMSE	TV-BN-PWFTS	317.2940	18.8346	44.6259	144.3049	344.3751	48.5997	0.0343	0.0134	0.0312
	TV-BN-PWFTS (NSFS)	94.7724	7.7763	27.8757	62.1150	156.4451	19.4565	0.0061	0.0032	0.0074
	TV-BN-PWFTS (adaptive)	93.8905	7.1645	28.2721	**56.5443**	141.0029	20.6079	**0.0060**	**0.0031**	0.0071
	TV-NS-BN-PWFTS	**92.9266**	**7.1490**	**27.5051**	57.7796	**138.6654**	**18.3194**	**0.0060**	**0.0031**	**0.0070**
MAPE	TV-BN-PWFTS	3.5785	1.3729	1.2272	1.2636	6.8562	9.2859	2.1052	1.2701	1.3969
	TV-BN-PWFTS (NSFS)	1.0378	0.5341	0.7658	0.5523	2.6283	3.5073	0.3450	0.2760	0.3203
	TV-BN-PWFTS (adaptive)	1.0215	**0.4828**	0.7659	**0.5012**	**2.5094**	3.4801	0.3409	0.2728	0.3160
	TV-NS-BN-PWFTS	**1.0174**	0.4881	**0.7534**	0.5120	2.5151	**3.4196**	**0.3402**	**0.2696**	**0.3114**
U	TV-BN-PWFTS	3.3514	2.3902	1.6019	2.2860	2.2215	2.4291	5.6060	4.1973	4.2130
	TV-BN-PWFTS (NSFS)	1.0065	0.9947	1.0047	0.9972	1.0325	1.0018	1.0040	1.0042	1.0021
	TV-BN-PWFTS (adaptive)	0.9972	0.9164	1.0190	**0.9077**	0.9306	1.0611	0.9873	**0.9741**	0.9714
	TV-NS-BN-PWFTS	**0.9870**	**0.9144**	**0.9913**	0.9275	**0.9151**	**0.9432**	**0.9845**	0.9745	**0.9581**

The bold values indicate the best performance for each metric on each dataset.

## Data Availability

The datasets’ sources are fully annotated in the paper.
